# Occipital Neuralgia after Hair Transplantation and Its Treatment

**DOI:** 10.1155/2015/428413

**Published:** 2015-01-26

**Authors:** Jason Siefferman, Yury Khelemsky

**Affiliations:** ^1^U.S. Department of Veteran's Affairs, Manhattan Campus, New York, NY 10010, USA; ^2^Icahn School of Medicine at Mount Sinai, Department of Anesthesiology, New York, NY 10029, USA

## Abstract

While undergoing full thickness tissue harvest from the posterior scalp, a 72-year-old man experienced immediate severe pain in the right occiput and was unable to complete the procedure. The pain was constant “sharp” and “shocking” with numbness in the distribution of the lesser occipital nerve, exacerbated by physical activity, and local anesthetic blocks provided temporary complete relief. After numerous treatments over several years, including oral analgesics, botulinum toxin injections, and acupuncture, proved ineffective, pulsed radiofrequency neuromodulation provided greater than 80% relief for 5 months.

## 1. Introduction

Occipital neuralgia (ON) is defined by the International Headache Society as a neuralgiform headache characterized by paroxysmal shooting or lancinating pain in the greater, lesser, or third occipital nerve dermatomes [1]. Pain is unilateral in 85% of patients, with the greater occipital nerve affected in 90% of cases [2]. ON is most commonly idiopathic, although it has also been associated with many processes including trauma, chronic muscle tightness [3], cervical spondylosis [4], giant cell arteritis [5], and many other processes [6].

One technique for hair transplantation surgery involves harvesting a full-thickness skin graft from the posterior scalp [7]. As the occipital nerves provide sensory innervation to this region and course just above the galea, the possibility of nerve injury during graft harvest exists. We report a case of occipital neuralgia associated with autologous hair transplantation.

## 2. Case Description

A 72-year-old man with androgenic alopecia experienced immediate severe pain in the right occiput while undergoing graft harvest from the posterior scalp. The pain was “sharp” and “shocking” with numbness and tingling extending up to the posterior right ear, consistent with the distribution of the lesser occipital nerve (LON).

When, after several months, his pain failed to improve with oxycodone, neuropathic agents, and acupuncture, he sought further care with a headache specialist. At that time his pain quality was unchanged, it was exacerbated by physical activity, and local anesthetic blocks provided only temporary, although complete, relief. He continued to have sensory deficits in the distribution of the LON and exacerbation of symptoms with tapping of the LON (Tinel's sign).

Pregabalin was initiated and titrated to the largest tolerated dose of 75 mg three times daily with modest improvement. Botulinum toxin type A (BTX) of 100 units was injected in a grid-like fashion over the right occiput, which also provided modest additional relief for 3 months. Follow-up BTX injections, however, were unhelpful.

**Figure 1:**
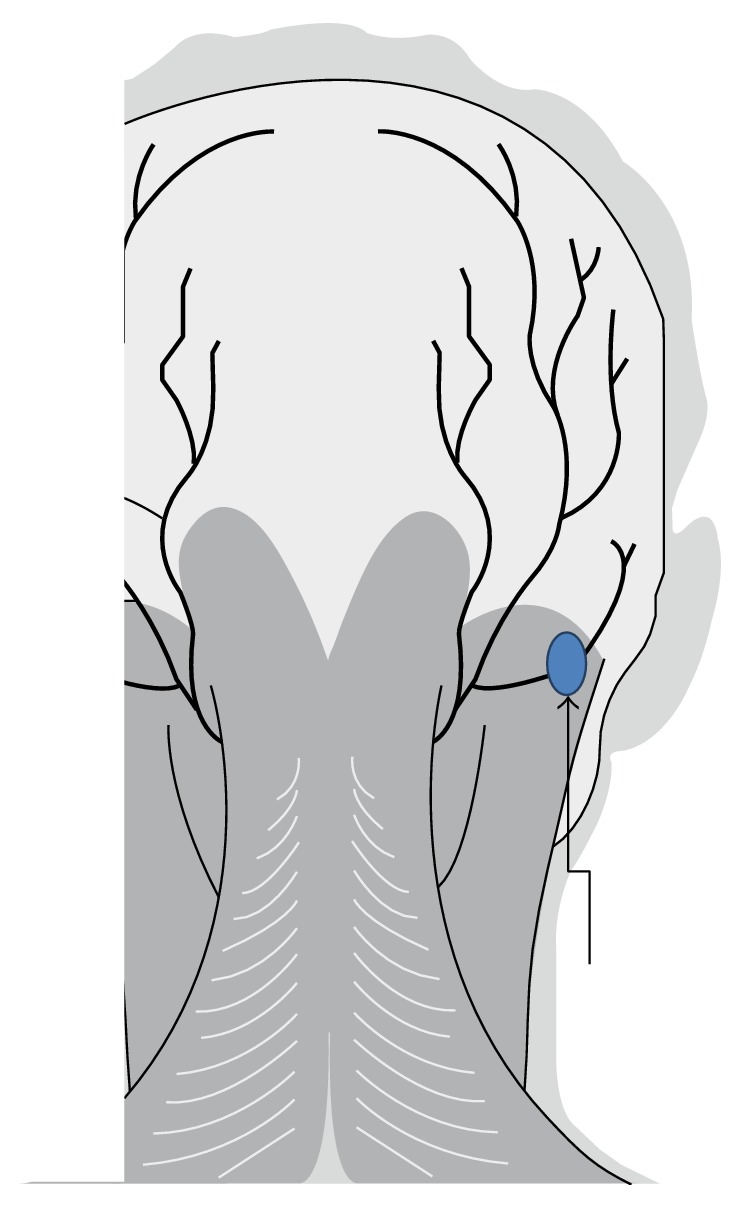
Oval represents point of maximal tenderness along the lesser occipital nerve. RF cannulae were positioned at the superior, inferior, medial, and lateral aspects of the point of maximal tenderness prior to pulsed RF.

He was then referred to a pain specialist for possible occipital nerve ablation. Repeat blocks of the occipital nerve confirmed its role in nociceptive process, and the patient agreed to undergo pulsed radiofrequency neuromodulation (PRF). With the patient in the prone position, a neuroma was identified at the point of maximal tenderness along the LON. Under conscious sedation, the area was anesthetized with bupivacaine 0.25% 3 mL with 1 : 200 K epinephrine. Two radiofrequency cannulae (5 cm/5 mm, NeuroTherm Inc., Wilmington, MA) were introduced at the superior aspect of the point of maximal tenderness, and pulsed radiofrequency at 42°C for 4 minutes was initiated. The cannulae were repositioned to the inferior, medial, and lateral aspects of the point of maximal tenderness with pulsed RF performed for 4 minutes at each position, for a total time of 16 minutes (Figure 1).

The patient reported 100% pain relief starting about 1 week after the procedure and continued to have greater than 80% pain relief for 5 months. The procedure was then repeated with similar success.

## 3. Discussion

Tissue for hair transplantation is commonly harvested as a narrow strip across the posterior scalp, which overlies one or more occipital nerves. In this case, nerve injury may have occurred as a direct transection or secondary to traction required for closure [7].

Treatment of ON may consist of oral medications, local anesthetic blocks, botulinum toxin [8], pulsed radiofrequency neuromodulation [9], radiofrequency ablation [10], cryoablation, peripheral nerve stimulation [11], or surgical decompression [12].

This patient experienced moderate relief with pregabalin and the initial botulinum toxin treatment and significant relief after pulsed radiofrequency neuromodulation.

## 4. Conclusion

Posterior scalp graft harvest for hair transplantation may result in injury to the occipital nerves leading to chronic neuropathic pain, which may be amenable to treatment with pulsed radiofrequency neuromodulation.
